# *Candida albicans* Ubiquitin and Heat Shock Factor-Type Transcriptional Factors Are Involved in 2-Dodecenoic Acid-Mediated Inhibition of Hyphal Growth

**DOI:** 10.3390/microorganisms8010075

**Published:** 2020-01-03

**Authors:** Dongliang Yang, Yanling Hu, Zixin Yin, Qianru Gao, Yuqian Zhang, Fong Yee Chan, Guisheng Zeng, Lixing Weng, Lianhui Wang, Yue Wang

**Affiliations:** 1 Jiangsu Key Laboratory for Biosensors, Institute of Advanced Materials (IAM), Nanjing University of Posts and Telecommunications, 9 Wenyuan Road, Nanjing 210046, China; yangdl1023@163.com (D.Y.); yinzixin18@163.com (Z.Y.); gaoqianru18@163.com (Q.G.); kekedemajiao@live.cn (Y.Z.); iamlhwang@njupt.edu.cn (L.W.); 2 School of Physical and Mathematical Sciences, Nanjing Tech University, Nanjing 211816, China; 3 School of Electrical and Control Engineering, Nanjing Polytechnic Institute, No. 625, Geguan Road, Nanjing 210048, China; huyanling@njpi.edu.cn; 4 Institute of Molecular and Cell Biology, 61 Biopolis Drive, Proteos, Singapore 138673, Singapore; fengyi.sky@gmail.com (F.Y.C.); mcbwangy@imcb.a-star.edu.sg (Y.W.); 5 College of Geography and Biological Information, Nanjing University of Posts and Telecommunications, Nanjing 210046, China

**Keywords:** *Candida albicans*, 2-dodecenoic acid, Ubi4, Sfl1, Sfl2

## Abstract

*Cis*-2-dodecenoic acid (i.e., *Burkholderia cenocepacia* Diffusible Signal Factor, BDSF), a signaling molecule produced by *Burkholderia cenocepacia* but not by *Candida albicans*, can prevent *Candida albicans* hyphal formation. The mechanism by which BDSF controls the morphological switch of *C. albicans* is still unknown. To address this issue, we used the cDNA microarray method to investigate the differential expression of genes in *C. albicans* in the presence and absence of BDSF. The microarray result indicated that 305 genes were significantly different in the expression level. This included the downregulation of 75 genes and the upregulation of 230 genes. Based on the microarray data, a mutant library was screened to search for genes, once mutated, conferred insensitivity to BDSF. The results showed that the repressors (Ubi4 and Sfl1 proteins) and the activator (Sfl2 protein) of filamentous growth are involved in the BDSF regulation of hyphal morphogenesis. Ubi4, an ubiquitin polypeptide that participates in ubiquitin-mediated protein turnover, is the protein required for the degradation of Sfl2. Sfl1 and Sfl2 proteins antagonistically control *C. albicans* morphogenesis. In the hyphal induction condition, the amount of Ubi4 and Sfl1 protein increased rapidly with the exogenous addition of BDSF. As a result, the protein level of the activator of filamentous growth, Sfl2, decreased correspondingly, thereby facilitating the *C. albicans* cells to remain in the yeast form.

## 1. Introduction

*Candida albicans* is a prevalent opportunistic fungal pathogen that is frequently observed in the mucosal surfaces of healthy individuals [1]. However, under favorable conditions, this organism can proliferate and cause superficial infections such as rash and thrush [2]. *C. albicans* can also disseminate via the bloodstream resulting in life-threatening systemic infections in immunocompromised individuals [3,4,5]. At present, *Candida* bloodstream infection ranks as the fourth most prevalent hospital-acquired bloodstream infection and according to the clinical pathogen diagnostic data, it is associated with increased morbidity and mortality rates [6].

Virulence factors of *C. albicans* (e.g., morphogenesis, secretion of degradative enzymes, and surface adhesion proteins) are closely related to its pathogenicity [2,7,8]. Among the virulence traits of *C. albicans*, the ability to switch growth forms between yeast, pseudohyphae, and hyphae is critically important [9]. This phenotypic plasticity is governed by many different environmental cues, including pH, temperature, nutrient availability, and the presence of chemicals that stimulate or repress the filamentation pathway [10,11]. The quorum-sensing molecule farnesol, produced by *C. albicans*, inhibits hyphal growth by downregulating cAMP/PKA signaling [2,12]. *Cis*-2-dodecenoic acid (BDSF) is a farnesol analogue that has been shown to be more effective in inhibiting *C. albicans* hyphal formation. It can be extracted from *Burkholderia cenocepacia* metabolite but not from *C. albicans* [13,14,15].

The Ras/cAMP/PKA and MAP kinase signaling pathways play a pivotal role in *C. albicans* morphogenesis [1]. Aside from these pathways, the activation and inhibition of hyphal development involves a combination of positive and negative regulation via multiple transcription factors [1,16]. Among them, the enhanced filamentous growth protein 1 (Efg1), acting downstream of the cAMP/PKA pathway, is crucial in *C. albicans* morphogenesis. Furthermore, Efg1 is required for the downregulation of Nrg1, a transcriptional repressor of hyphal morphogenesis [17]. Sfl1 is another transcriptional factor that is downstream of the cAMP/PKA pathway. Sfl1 is a heat shock factor-type transcriptional regulator that negatively regulates filamentation. Deletion of *SFL1* leads to flocculation, hyperfilamentation, and hyphae-specific gene expression in several conditions [18,19,20]. Interestingly, Sfl2 and Sfl1 are structural homologs of *Saccharomyces cerevisiae* Sfl1 and functionally complement a *S. cerevisiae sfl1∆/∆* mutant [16,19], but Sadri et al. found that Sfl2 and Sfl1 of *C. albicans* have antagonistic functions and serve as central “switch on/off” regulators to control the morphogenesis [21]. Overexpression of Sfl2 can upregulate hyphae-specific genes and activate hyphal growth [16].

Ubiquitin-mediated protein turnover is an essential regulatory mechanism that is involved in numerous physiological and pathological processes, including signal transduction, differentiation, development, and cancer therapy [12,22]. In *C. albicans*, *UBI4* encodes an ubiquitin polypeptide that contains three ubiquitin tandem repeats and participates in the negative control of morphogenesis [23]. Previous study indicates that farnesol inhibits hyphal initiation by blocking the ubiquitin ligase (Ubr1) mediated degradation of the transcriptional repressor Cup9 [12]. Other ubiquitin ligases (Cdc4 and Rad6) are also involved in *C. albicans* morphogenesis through mediating target protein degradation [24,25]. These results illustrate that the ubiquitin-proteasome system plays a vital role in *C. albicans* morphogenesis [26].

In this study, a cDNA microarray analysis was implemented to probe gene expression changes in *C. albicans* in the presence and absence of BDSF. Then a mutant library was constructed based on the microarray data. By screening the mutant library, we identified *ubi4∆/∆* and *sfl1∆/∆* mutants as being insensitive to BDSF. We also found that a strain overexpressing *SFL2* is resistant to BDSF. The gene expression patterns of *UBI4*, *SFL1* and *SFL2* in the presence or absence of BDSF were also investigated by Western analysis. In *ubi4∆/∆* mutant, an abnormal protein expression level of Sfl1 and Sfl2 was observed, suggesting that ubiquitin-mediated degradation is critical for the regulation of Sfl1 and Sfl2 content in *C. albicans*.

## 2. Materials and Methods

### 2.1. Strains and Medium

The *C. albicans* strains used in this study are listed in Table 1. Several strains were kindly provided by Wang Yue (WY) lab from Institute of Molecular and Cell Biology, Singapore. BWP17 is the parental strain for the construction of other strains. BDSF was synthesized as described [27]. A stock solution of 0.3 M BDSF was prepared in ethanol and diluted with YPD medium (1% Difco yeast extract, 2% Difco peptone, and 2% dextrose) supplemented with 5% fetal bovine serum (FBS) (Thermo Fisher, VIC, Australia). Yeast cells were grown at 30 °C in YPD or GMM medium (2% glucose and 0.67% Difco yeast nitrogen base). For hyphal induction, *C. albicans* cells were scraped from the YPD plate, resuspended in YPD medium supplemented with 5% FBS (about 1 × 10^5^ cells mL^−1^), and incubated at 37 °C in the presence or absence of 150 μΜ BDSF. In the control group, 150 μΜ ethanol was used.

### 2.2. Microarray Analysis

Fresh *C. albicans* cells were scraped from YPD plates and hyphal induction was conducted in YPD supplemented with 5% FBS and incubated at 37 °C for 4 h in the presence of 150 μΜ BDSF or ethanol (control). After confirming the hyphal formation of control cells under microscope, cultures were transferred to a 1.5 mL tube and cells were harvested by centrifugation (8000 rpm, 8 min). Total RNA was extracted using the EASYspin yeast kit (Aidlab, Beijing, China) according to the manufacturer’s procedure. The quality of RNA was evaluated using an Agilent bioanalyzer 2100 (Agilent Technologies, Palo Alto, CA, USA). The microarray experiments were carried out at the bioassay laboratory of CapitalBio Corporation (Beijing, China) using the 8 K *C. albicans* genome array. In order to estimate the gene expression difference between BDSF- and ethanol-treated cells, gene expression in BDSF-treated cells was compared to *C. albicans* cells treated with 150 μΜ ethanol by hybridizing differentially labeled samples of each for the same array. Hybridized arrays were analyzed using a LuxScan 10 KA confocal laser scanner (CapitalBio Corp., Beijing, China). Then Excel and GeneSpring software (Silicon Genetics, Redwood, CA, USA) was used to conduct gene expression difference analysis. Differences in gene expression between the experimental and reference samples were considered significant when the value was ≥2 or ≤0.5 (*p* ≤ 0.05).

### 2.3. Gene Deletion and Tagging

Gene disruption cassettes were generated by flanking either the *URA3* (U) or *HIS1* (H) marker with DNA fragments that amplified from the 5′ and 3′ flanking regions of the target gene, respectively [28]. The disruption cassettes were then transformed into *C. albicans* BWP17 to construct gene deletion mutants. Successful gene disruptions were verified by colony PCR as described previously [29]. To obtain the rescue mutant, the whole coding sequence of the target gene with its native promoter was amplified and cloned into the CIp10 vector with *ARG4* (A) marker and a C-terminal GFP (Green Fluorescence Protein) tag [29]. The plasmid was then linearized with the *Asc*I enzyme (New England Biolabs, Beverly, MA, USA) and transformed into the deletion mutant using the Fast™ Yeast Transformation Kit (G-Biosciences, Maryland Heights, MO, USA). The transformants were verified by GFP fluorescence signal detection or Western blotting. To tag *SFL1* and *SFL2* at the C terminus with *Myc* or *HA*, the C terminal region of *SFL1* and *SFL2* was amplified, and cloned into the CIp10 vector containing either *Myc* or *HA* tag [30]. The resulting plasmid was then linearized by digestion with an enzyme located within the gene fragment and transformed into the recipient strain. The transformants were verified by Western blotting. To generate a *SFL2* overexpression construct, the entire open reading frame of *SFL2* except stop code was amplified and used to replace RHO1_900_ in the plasmid Tetoff-Myc-RHO1_900_-UTR^*^-TetR/CIP10-URA3 (available from our laboratory). The resulting construct was linearized with the *Asc*I enzyme (New England Biolabs, Beverly, MA, USA) and transformed into the BWP17 strain. Finally, Western blot was used to select the transformants that overexpressed Sfl2 protein.

### 2.4. Microscopic Assessment of C. albicans Strains Hyphal Formation

To screen the BDSF-resistant mutants, *C. albicans* strains were grown on YPD plates overnight at 30 °C, then the cells were inoculated into YPD medium containing 5% FBS supplement with or without 150 µM BDSF. After a 4 h incubation at 37 °C on a rotary mixer (Scilogex, Redwood, CA, USA) in a 1.5 mL tube, cells were pelleted and their morphologies were examined with a microscope. The percentage of hyphal formation was calculated by the number of hyphae versus the total number of *C. albicans* cells. About 200 cells were counted in each sample.

### 2.5. Protein Extraction and Western Blotting

Protein extraction was performed as previously described [31]. In brief, cells were harvested and an equal volume of acid-washed glass beads (Sigma, St. Louis, MO, USA) was added. Next, 500 μL of urea lysis buffer (54% urea, 0.476% N-2-hydroxyethylpiperazine-N-2-ethane sulfonic acid, 0.11% sodium acid pyrophosphate, and 0.18% sodium orthovanadate) was added and followed by four rounds of 60 seconds homogenization in a MicroSmash MS-100 beater (TOMY, Tokyo, Japan) with 1 min intermission between rounds. After centrifugation at 14,000 rpm for 10 min, the supernatants were collected and mixed with 3x protein loading buffer (150 mM Tris-HCl [pH 6.8], 300 mM DTT, 6% SDS, 0.3% Bromophenol blue, 30% Glycerol). For direct Western blotting, the protein samples were separated by 10% or 12% sodium dodecyl sulfate-polyacrylamide gel electrophoresis (SDS-PAGE) and transblotted to a polyvinylidene difluoride (PVDF) membrane (Bio-Rad, Hercules, CA, USA). The membrane was blocked with 5% milk for at least 1 h, then washed with PBS-T (Phosphate-Buffered Saline, 0.1% Tween 20) for 3 times (5 min each), followed by incubation with mouse monoclonal Myc, HA, or GFP antibodies (Santa Cruz Biotechnology, Santa Cruz, CA, USA) for 1 h. After 3 washes with PBS-T, the membrane was further incubated with horseradish peroxidase (HRP) conjugated anti-mouse IgG antibody (Santa Cruz Biotechnology, Santa Cruz, CA, USA) for 1 h. Finally, the membrane was washed for times and the target protein was detected using an Electrochemiluminescence (ECL) Western Blotting Detection Kit (GE Healthcare, Amersham, UK). Protein bands on Western blot were analyzed using ImageJ software (https://imagej.nih.gov/ij/).

## 3. Results

### 3.1. BDSF Inhibits Filament Formation in C. albicans

**Figure 1:**
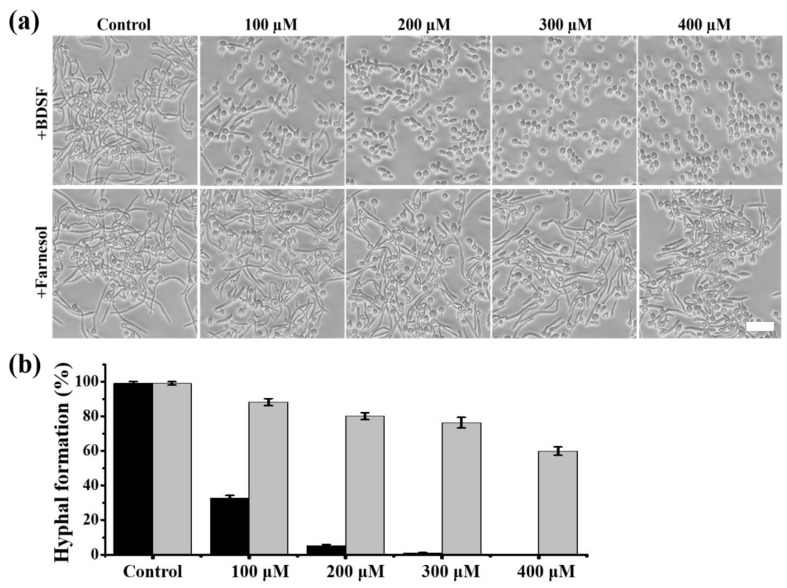
(**a**) Hyphal formation was induced in YPD medium (1% Difco yeast extract, 2% Difco peptone, and 2% dextrose) + 5% fetal bovine serum (FBS) with different concentrations of *Cis*-2-dodecenoic acid (BDSF) or farnesol at 37 ℃ for 4 h. (**b**) Effects of different concentrations of BDSF (black) or farnesol (grey) on *C. albicans* hyphal formation. Bar, 15 µm.

Our previous study indicated that *C. albicans* filament formation is more effectively inhibited by exogenous addition of BDSF than farnesol in glucose minimum medium (GMM) containing 20% filtered FBS or RPMI 1640 supplemented with 4-morpholinepropanesulfonic acid [32,33]. At present, *C. albicans* remains the main species of *Candida* bloodstream infections [28,34]. We therefore checked the inhibitory effect of BDSF on hyphal formation induced by the whole FBS. As shown in Figure 1, BDSF inhibits *C. albicans* hyphal growth in a dose-dependent manner and has a greater inhibitory activity than farnesol. The hyphal growth inhibition rate was increased from 67.3% to 94.9% when the concentration of BDSF increased from 100 μM to 200 μM. On the contrary, when the concentration of farnesol increased from 100 μM to 400 μM, the hyphal growth inhibition rate was increased from 11.8% to 40.2% (Figure 1b). These results indicate that BDSF possesses a superior ability of inhibiting hyphal growth.

### 3.2. Differential Gene Expression in C. albicans Treated with BDSF

**Figure 2:**
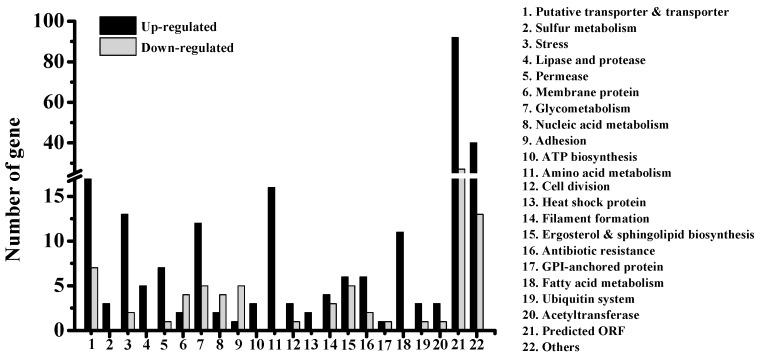
Functional classification of *C. albicans* genes regulated by BDSF (150 μM) treatment.

To investigate the molecular mechanism by which BDSF inhibits filament formation, we next conducted cDNA microarray analysis to examine the altered gene expression profile of *C. albicans* treated with BDSF. The microarray data of this experiment are available from the Gene Expression Omnibus (GEO) database under accession number GSE137546. Comparison between BDSF- and ethanol-treated cells revealed that 305 genes were significantly affected on the expression level by BDSF treatment. These genes were classified according to their biological functions (http://www.candidagenome.org/). We found that these BDSF-modulated genes are associated with a variety of biological functions including hyphal formation, cell wall structure, adherence, antibiotic resistance, transportation, cell membrane permeabilization, ergosterol biosynthesis, glucose metabolism, and fatty acid metabolism. As shown in Figure 2, seven genes related to filament formation were differentially expressed. Among them, *CHK2*, *orf19.5576*, *ERG3*, and *SFL2* are required for filamentous growth [35,36]. Downregulation of these genes may therefore block filament formation. *UBI4* encodes an ubiquitin precursor and disruption of *UBI4* promotes hyphae and pseudohyphae formation under certain circumstances [37,38]. *SFL1* encodes a negative regulator of hyphal development. Thus, upregulation of *UBI4* and *SFL1* may inhibit filament formation. Currently, most commercial antifungals target the ergosterol biosynthesis pathway by, for example, blocking ergosterol synthesis or selectively binding to ergosterol to further disrupt membrane integrity [39]. *ERG1*, *ERG3*, and *ERG11* encode enzymes necessary for ergosterol synthesis [40]. *UPC2* encodes a Zn2-Cys6 transcript factor that upregulates *ERG2* and *ERG11* expression. The *UPC2* disruption mutant shows reduced ergosterol level and high susceptibility to antifungals [41]. *SUR2* encodes ceramide hydroxylase, which is needed for sphingolipid biosynthesis. Both ergosterol and sphingolipid are involved in membrane polarization and hyphal morphogenesis in *C. albicans* [42]. Therefore, downregulation of the genes of the sphingolipid and ergosterol biosynthetic pathway may result in cells maintaining in yeast form. BDSF treatment also resulted in the upregulation of several genes encoding cell cycle proteins, DNA repair enzymes, and heat shock proteins. This was also observed in *C. albicans* biofilm treated with farnesol [43].

### 3.3. Screening for BDSF-Resistant Mutants

**Figure 3:**
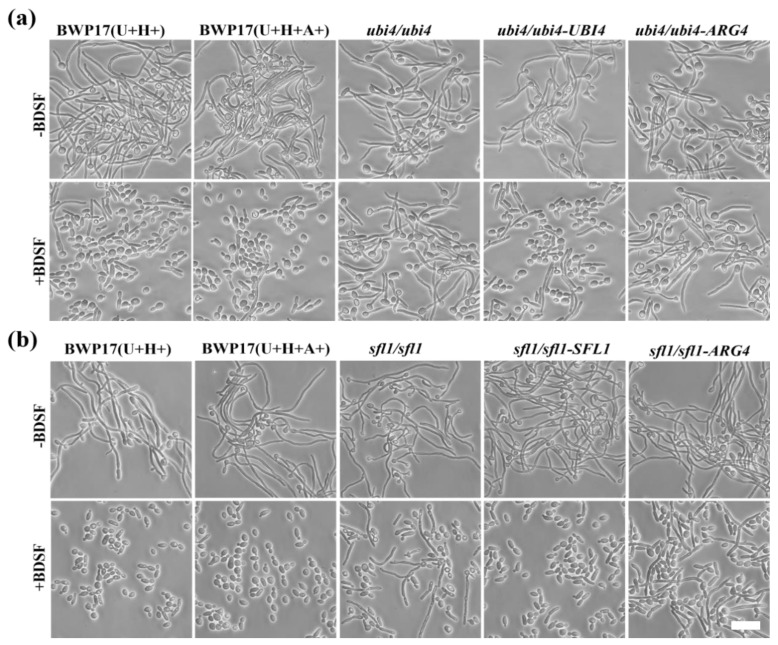
(**a**) *ubi4∆/∆* and (**b**) *sfl1∆/∆* mutants exhibited BDSF resistance. Cells were cultured in YPD medium and induced for hyphal formation with 5% FBS at 37 °C for 4 h in the presence or absence of 150 μM BDSF. Bar, 15 µm.

To verify the findings deduced from the microarray analysis, we chose genes involved in filament growth and regulation for further examination. We knocked out each of the genes to construct a mutant library. Each mutant strain was cultured in YPD medium and induced for hyphal growth with 5% FBS at 37 °C for 4 h in the presence or absence of 150 μM BDSF. Both *ubi4∆/∆* and *sfl1∆/∆* mutants exhibited insensitivity to BDSF and continued to produce hyphae in the presence of BDSF. In contrast, cells of wild-type control BWP17(U+H+) largely remained in yeast form under the same condition (Figure 3a,b). To further confirm that *UBI4* and *SFL1* are truly the genes required for BDSF resistance, wild-type *UBI4* and *SFL1* genes were introduced into the corresponding deletion mutants to generate rescue strains. Similar to the wild-type control BWP17(U+H+A+), both rescue strains (*ubi4/ubi4-UBI4* and *sfl1/sfl1-SFL1*) regained the sensitivity to BDSF and failed to produce normal hyphae, while mutants transformed with the vector (*ubi4/ubi4-ARG4* and *sfl1/sfl1-ARG4*) maintained the resistance to BDSF (Figure 3a,b).

**Figure 4:**
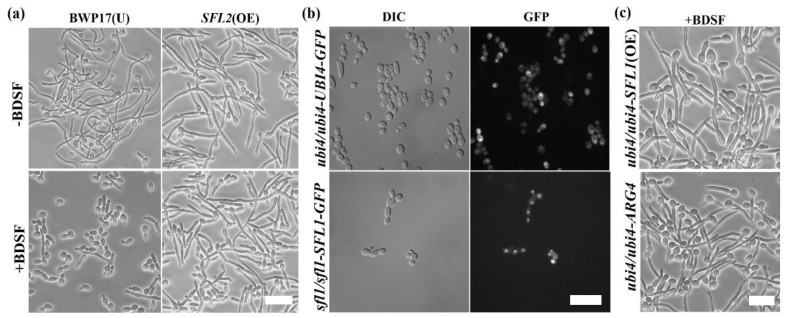
(**a**) Overexpression (OE) of *SFL2* leads to BDSF resistance. (**b**) Subcellular localization of SFL1-GFP and UBI4-GFP in *C. albicans* cells. (**c**) Overexpression of *SFL1* in *ubi4∆/∆* mutant remained insensitive to BDSF. Hyphal induction was performed with the addition of 5% FBS and maintained at 37 °C for 4 h in the presence or absence of 150 μM BDSF. Bars, 15 µm.

In *S. cerevisiae*, both *CaSFL1* and *CaSFL2* can complement the loss of *ScSFL1*, indicating that these two genes perform similar functions in *S. cerevisiae.* However, *CaSFL1* and *CaSFL2* have antagonistic functions in *C. albicans* morphogenesis [21]. In support of this finding, we found that cells overexpressing *SFL2* conferred resistance to BDSF similar to the *SFL1* deletion cells under the same condition (Figure 4a). Previous studies indicated that Ubi4 is located in the cytoplasm and Sfl1 is located in the nucleus [20,44]. We further confirmed the results by examining the subcellular localization of GFP-tagged Ubi4 and Sfl1 (Figure 4b). Based on their subcellular localization, it is possible that *SFL1* may act downstream of *UBI4* to regulate filament formation. However, as shown in Figure 4c, overexpression of *SFL1* in *ubi4∆/∆* mutant failed to reduce the resistance of *ubi4∆/∆* mutant to BDSF, suggesting that *SFL1* acts in a pathway different from that of *UBI4*.

### 3.4. The Change in Expression Level of Proteins

**Figure 5:**
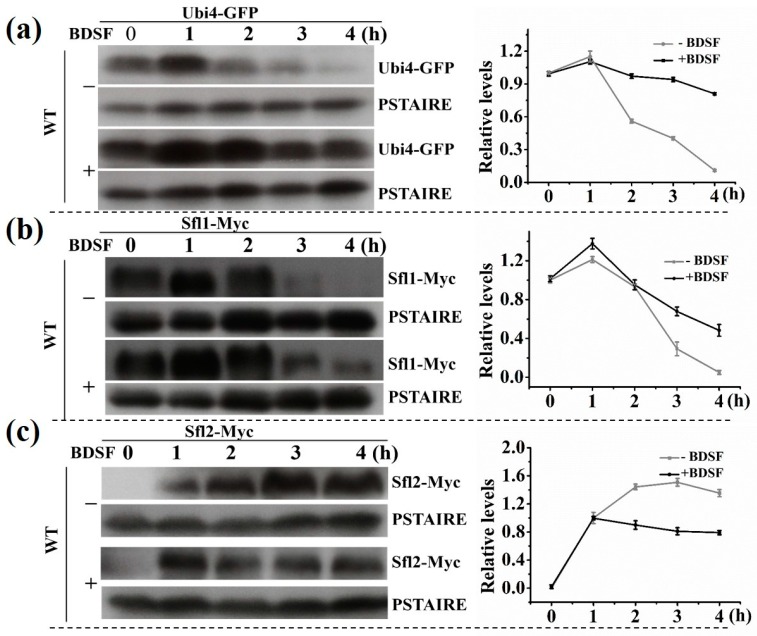
Hyphal growth of wild-type (WT) cells carrying (**a**) Ubi4-GFP, (**b**) Sfl1-Myc, and (**c**) Sfl2-Myc was induced with 5% FBS at 37 °C in the presence or absence of 150 μM BDSF. Samples were taken at 1 h intervals for Western blot analysis (left panels). Relative protein expression levels (right panels) were analyzed using ImageJ software. Normalizations were performed against the corresponding protein signal at 0 h (**a**,**b**) or 1 h (**c**).

According to the *Candida* genome database, Sfl1 is a transcription factor that represses hyphal morphogenesis. In contrast, Sfl2 is a transcription factor that is required for hyphal morphogenesis. *UBI4* encodes an ubiquitin precursor that participates in the management of protein concentration [45]. To examine the protein level of Ubi4, Sfl1, and Sfl2 during hyphal growth, we tagged each of the proteins with GFP or Myc at the C terminus, respectively. Upon hyphal induction (without BDSF), the level of Ubi4 and Sfl1 slightly increased initially, then rapidly decreased following the extension of hyphal induction time (Figure 5a,b). Sfl2, as an activator of hyphal growth, was upregulated in this process (Figure 5c). In the presence of BDSF, the protein level of Ubi4 and Sfl1 decreased in a much slower rate; but the protein level of Sfl2 began to drop rapidly after an initial increase (Figure 5a–c).

**Figure 6:**
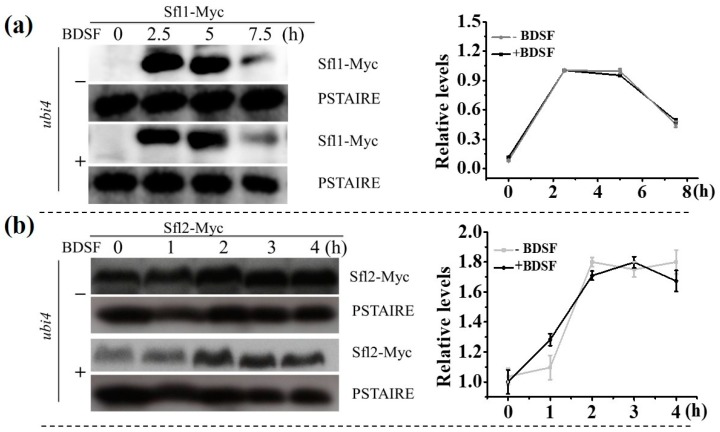
*ubi4* mutant cells expressing (**a**) Sfl1-Myc or (**b**) Sfl2-Myc were induced for hyphal growth with 5% FBS at 37 °C in the presence or absence of 150 μM BDSF. Samples were taken for Western blot analysis at the time points as indicated (left panels). Relative protein expression levels (right panels) were analyzed using ImageJ software. Normalizations were performed against the corresponding protein signal at 2.5 h (**a**) and 0 h (**b**), respectively.

In order to investigate the differential expression of *SFL1* and *SFL2* in *ubi4∆/∆* mutant, both Sfl1 and Sfl2 in the mutant were tagged with Myc at the C terminus. Deletion of *UBI4* showed very little effect on the protein expression pattern of Sfl1 (Figure 5b and Figure 6a) and Sfl2 (Figure 5c and Figure 6b) during the hyphal growth in the absence of BDSF. With the presence of BDSF, the protein level of Sfl1 decreased slightly faster in *ubi4∆/∆* mutant cells (Figure 5b) than in wild-type cells (Figure 6a). However, in the presence of BDSF, the rapid decrease of Sfl2 protein amount seen in wild-type cells (Figure 5c) was mostly inhibited and remained in a high level (Figure 6b), indicating that Ubi4 may mediate the degradation of Sfl2.

**Figure 7:**
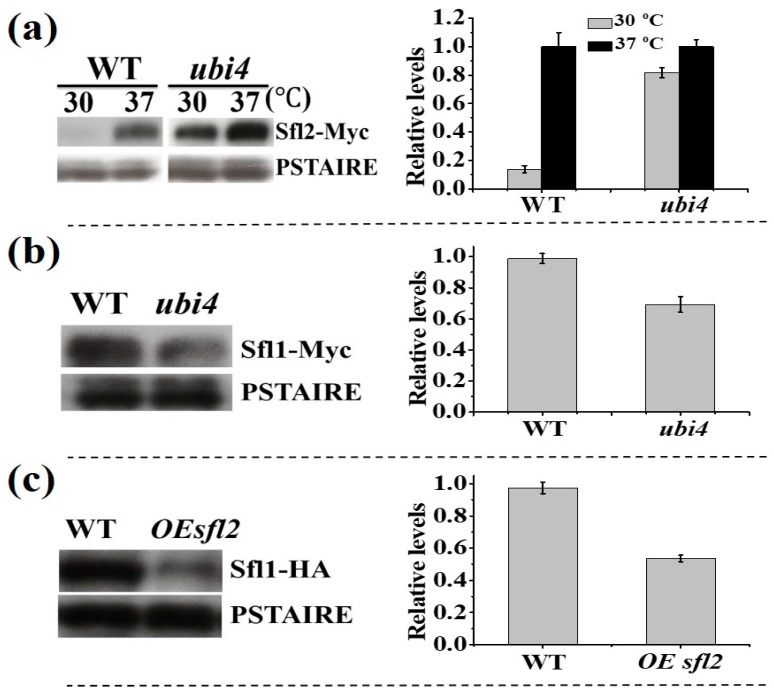
(**a**) WT and *ubi4* mutant cells expressing Sfl2-Myc were first grown at 37 °C in GMM medium (2% glucose and 0.67% Difco yeast nitrogen base) for 4 h, then shifted to 30 ℃ and cultured overnight. (**b**) WT and *ubi4* mutant cells expressing Sfl1-Myc were cultured overnight in GMM at 30 °C. (**c**) WT and *SFL2* overexpression cells expressing Sfl1-HA were cultured at 30 °C overnight. Right panels show corresponding protein relative expression level. Protein bands on Western blot (left panels) were analyzed using ImageJ software. Normalizations were performed against the corresponding protein signal at 37 °C (**a**) or in wildtype cells (**b**,**c**).

To further confirm that Ubi4 is required for Sfl2 degradation, wildtype and *ubi4∆/∆* mutant cells were cultured at 37 °C for 4 h to induce Sfl2 expression, then transferred to 30 °C and further cultured overnight. As shown in Figure 7a, Sfl2-Myc was detected in both strains when cells were grown at 37 °C. When the temperature was changed to 30 °C, Sfl2 protein could only be detected in the *ubi4∆/∆* mutant. This result further indicated that Ubi4 is necessary for Sfl2 degradation. Christophe et al. found that Sfl1 and Sfl2 act as a morphology control center and antagonistically control *C. albicans* morphogenesis [21]. In contrast to Sfl2 whose protein level was higher in *ubi4∆/∆* mutant cells than in wild-type cells (Figure 7a), We found that Sfl1 had lower protein level in *ubi4∆/∆* mutant cells than in wild-type cells (Figure 7b). We also found that Sfl1 protein level was reduced when the cells overexpressed *SFL2* (Figure 7c). Based on the above results, we hypothesized that, in wild-type cells under hyphal inducing condition, BDSF treatment helps to maintain Ubi4 and Sfl1 proteins at a high level, resulting in rapidly decrease of Sfl2 protein, hence inhibits hyphal morphogenesis.

## 4. Discussion

BDSF is an unsaturated fatty acid that is secreted by *B. cenocepacia* [32]. As a cross-kingdom signal molecule, BDSF regulates virulence of *B. cenocepacia* and activates the quorum-sensing system of *Xanthomonas campestris*. Furthermore, BDSF also suppresses the germination of *C. albicans* [13,32,33]. Compared to farnesol, BDSF is a more effective inhibitor of *C. albicans* hyphal morphogenesis. However, the mechanisms by which BDSF regulates hyphal formation remain to be elucidated. According to cDNA microarray analysis, a number of genes were differentially expressed when *C. albicans* cells were exposed to BDSF. We used gene knockout technology to confirm the functions of those genes that were involved in the BDSF response. We found that *UBI4*, *SFL1*, and *SFL2* play a crucial role in the BDSF regulation of hyphal morphogenesis. Deletion of *UBI4* completely abolished *C. albicans* response to BDSF, but the mutant cells regained sensitivity to BDSF when *UBI4* was ectopically expressed. This phenomenon suggested that Ubi4 is required for BDSF to block hyphal formation. In support of this hypothesis, we found that Ubi4 protein level was upregulated upon BDSF treatment. Consistently, downregulation of *UBI4* has been shown to promote filament formation [38]. The ubiquitin-mediated proteolytic degradation system has been known to implicate in the regulation of signal transduction networks that control filamentous growth [12]. For example, Lu et al. found that Ubr1-mediated degradation of Cup9 helps farnesol control hyphal initiation [12]. Atir-Lande and Roig et al. discovered that ubiquitin depletion results in expression level changes of genes associated with morphogenesis regulation [23,24].

Sfl1 and Sfl2 are transcription factors that act antagonistically to control morphogenesis in *C. albicans* [21]. *SFL1* and *SFL2* encode a transcriptional repressor and a transcriptional activator of hyphal formation, respectively [21]. *C. albicans* germ tube formation was inhibited under hyphal-inducing conditions when coincubated with *B. cenocepacia*, while in the *sfl1* deficiency mutant the hyphal growth was normal [44]. In this work, we further confirmed that Sfl1 is needed for BDSF to inhibit *C. albicans* hyphal morphogenesis. In the presence of BDSF, *C. albicans* remained in yeast form even in the hyphal induction condition. Under yeast culture condition, Sfl1 had higher protein amount in wild-type than in the *ubi4* mutant. In contrast, Sfl2 had lower protein amount in wild-type than in the *ubi4* mutant. In addition, overexpression of *SFL2* resulted in a reduction of Sfl1 protein level. Based on these results, we hypothesize that BDSF treatment may cause Sfl1 protein to remain at a relatively high level, and as a suppressor, Sfl1 may bind to other transcriptional activators of hyphal formation to inhibit hyphal formation.

Previous works indicated that Sfl1 and Sfl2 both localize to the nucleus in yeast and hyphal cells [16,20], while Ubi4 is found in the cytoplasm. Ubiquitin-mediated protein degradation plays a vital role in the morphological switch of *C. albicans.* The polyubiquitination-deficient mutant may cause an alteration in DNA arrangement and chromatin structure, further altering the expression of genes related to colony morphology, such as *TUP1* and *EFG1* [23]. Indeed, we found that the expression of genes involved in morphology control was changed in the *ubi4* deletion mutant. In general, Sfl2 cannot be detected when cells were cultured at 30 °C [16], but it could be detected in the *ubi4* mutant under the same condition. This result suggested that Ubi4 is needed for Sfl2 degradation.

## 5. Conclusions

In summary, DNA microarray analysis was used to identify the differentially-expressed genes between cells growing in hyphal-inducing condition in the absence or presence of BDSF. Subsequent mutant screens confirmed that three characterized genes were involved in the BDSF regulation of filament formation. With the presence of BDSF, two repressors of filamentous growth (Ubi4 and Sfl1) were maintained at a relative high level, and as a result, the morphological transition from yeast to hyphae was blocked. In addition, Ubi4 is the protein required for the degradation of Sfl2. High level of Ubi4 protein contributed to the degradation of Sfl2 (an activator of filamentous growth), thereby maintaining the *C. albicans* cells in the yeast form.

## Figures and Tables

**Table 1:** *C. albicans* strains used in this study.

Strain	Relevant Genotype	Source
BWP17	*ura3*::*imm434/ura3*::*imm434 his1*::*hisG/his1*::*hisG arg4*::*hisG/arg4*::*hisG*	WY lab
BWP17(A+)	*ARG4*	WY lab
BWP17(U+)	*URA3*	WY lab
BWP17(U+H+)	*URA3, HIS1*	WY lab
BWP17(U+A+)	*URA3, ARG4*	WY lab
BWP17(H+A+)	*HIS1, ARG4*	WY lab
BWP17(U+H+A+)	*HIS1, ARG4, URA3*	WY lab
WLX 11	*ubi4*Δ::*HIS1/ubi4*Δ::*URA3*	This study
WLX 12	*ubi4*Δ::*HIS1/ubi4*Δ::*URA3, ARG4*	This study
WLX 13	*ubi4*Δ::*HIS1/ubi4*Δ::*URA3, UBI4-GFP-ARG4*	This study
WLX 19	*sfl1*Δ::*HIS1/sfl1*Δ::*URA3*	This study
WLX 20	*sfl1*Δ::*HIS1/sfl1*Δ::*URA3, ARG4*	This study
WLX 21	*sfl1*Δ::*HIS1/sfl1*Δ::*URA3, SFL1-GFP-ARG4*	This study
WLX 60	*SFL1-HA-ARG4, SFL2-Myc (overexpression)-* *URA3*	This study
WLX 67	*SFL1-HA-ARG4*	This study
WLX 68	*sfl1Δ::HIS1/SFL1-Myc-ARG4*	This study
WLX 70	*ubi4*Δ::*HIS1/ubi41*Δ::*URA3, SFL1-Myc (overexpression)-ARG4*	This study
WLX 71	*ubi4*Δ::*HIS1/ubi41*Δ::*URA3, SFL2-Myc-ARG4*	This study
WLX 72	*SFL2-Myc::ARG4*	This study
